# Medicines, Morals and Manifestoes

**Published:** 1919-01-04

**Authors:** 


### The Hospital

The Workers' Newspaper of Administrative Medicine and Institutional Life, Administration, National Insurance and Health.

No. 1700, Vol. LXV. SATURDAY, JANUARY 4, 1919. PRICE TWOPENCE

### MEDICINES, MORALS AND MANIFESTOES.

For some reason not easy to define, the public in recent years have every now and again been treated to a manifesto signed by a number of medical practitioners claiming various decorations and distinctions. Alcohol was one of the earliest topics, and here the assertions of the first document provoked the appearance of a counter-blast supported, as was the original, by names bearing many alphabetical ornaments. Next came something termed "standard bread," and the purveyors of this article were enabled to decorate their shop windows with announcements supported by the appearance of much learning. Again, but a few weeks ago, the heads of several venerable medical organisations felt compelled to announce their opinion that the use of poisonous gases in warfare ought to be abolished—by treaty; and the wonder is that they did not say "by prescription." And now, as may be seen in the *Times* of December 28, the turn of venereal diseases has arrived. Whether this latest issue of collective wisdom will prove more effective than its predecessors time alone can tell, but remembering the fate of these omens are hardly auspicious.

The starting-point of the document is the danger to the public health which, it is alleged, will be produced by the spread of venereal infection to be expected on the demobilisation of our troops. Accepting this as beyond challenge, the statement is next made that no provision exists to meet the danger except "timid and hesitating counsels" and "weak and ineffective action." The National Council, which came into existence for the purpose of preventing these diseases, is told plainly that it "has failed," and that its failure is due to a non-recognition of the truth that "extraneous considerations can have no place in sanitary problems." It is held, in short, that the prevention of venereal diseases is a strictly parallel issue to the prevention of small-pox or typhoid, and that the question is one, not of morals, but of medicine. Further, the manifesto proclaims that these diseases can most successfully be brought under control by the adoption of "simple sanitary measures," and that the materials necessary for the application of these measures "can be obtained from any chemist." All these propositions are very precise, but some of them will hardly pass without challenge. It is at least possible that the National Council may have something to say in defence of its policy, and perhaps the taxpayer who was recently assured that salvation was to be found in "treatment centres," may intervene in the debate. It seems therefore probable that we shall have a further edition of the controversy whether individuals and the public generally should or should not be instructed in methods by which immoral and anti-social sexual acts can be committed without risk of penalty. The memorialists argue for this instruction, and they evidently regard the moral relations of the subject as an "extraneous consideration." But plainly this is to beg the question, for their opponents will hold that the influence upon private and public morals is one of the vital points in the problem. Both sets of controversialists will agree that what is "extraneous" ought to be excluded, but they will differ as regards the contents to be included in this term. In such difference lies really the heart of the present controversy. It is not a debate on the medical value of a certain policy, but on the morality of this policy, and distinguished professors and learned doctors do not help the situation by saying arbitrarily that the moral issue is "extraneous." On the contrary, and as a matter of fact, this is the main consideration that, rightly or wrongly, has always been urged against the policy which the *Times'* manifesto advocates, and such a consideration cannot be settled by doctors, who, indeed, have no special authority in the department of morals. If the gentlemen signing the manifesto wish to succeed they must convince public opinion that their policy is not only efficacious, but is also ethically justifiable. To stand on a medical pinnacle and to denounce the moral aspects of the debate as "extraneous" may have an authoritative note, but it has no real foundation in fact. This awkward point returns again and again, and until it is effectively met the manifesto will miss its aim.

Upon the evils and upon the mechanical method of meeting it, the manifesto is both precise and confident. But it becomes somewhat vague and shaky when the time for actual application is reached. The preventive agents exist and are easily accessible, and they should be "made known . . . by organised instruction." By whom is this instruction to be conveyed? This is surely a vital question, yet to it the manifesto affords no hint of a reply. Nor do the writers, with the opportunity of the *Times* at their disposal, tell us what are these simple measures that any chemist can supply. Why this silence if the question stands on the same level as the prevention of small-pox? In the latter case the "simple measures" would have been defined, and clergy, parents, teachers, and Press would have been urged to give them all possible publicity. Why do the memorialists pursue a different course in regard to the prevention of venereal disease if, as they contend, the question is nothing more than a "sanitary problem"? Their silence on the practical point is a conspicuous denial of their contention that the moral aspects of the instruction they advocate is an "extraneous consideration." At the moment we are not concerned with the question whether this instruction can or cannot be defended on the ground of morals. Our point is that this aspect of the matter cannot summarily be dismissed from the debate, and we believe that until the memorialists recognise this they are merely shutting their eyes to the chief of the difficulties which confront them.

